# A novel 3D-printed laryngoscope with integrated working channels for laryngeal surgery

**DOI:** 10.3389/fsurg.2023.906151

**Published:** 2023-03-14

**Authors:** Linus L. Kienle, Leon R. Schild, Felix Böhm, Rene Grässlin, Jens Greve, Thomas K. Hoffmann, Patrick J. Schuler

**Affiliations:** ^1^ Department of Otorhinolaryngology, Head and Neck Surgery, Ulm University Medical Center, Ulm, Germany; ^2^ Surgical Oncology Ulm, i2SOUL Consortium, Ulm, Germany

**Keywords:** laryngeal cancer, video endoscopy, 3D-printing, flexible instruments, laryngeal surgery, TORS

## Abstract

**Background:**

For the surgical treatment of early-stage laryngeal cancer, the use of transoral laser microsurgery (TLM) has emerged as the gold standard. However, this procedure requires a straight line of sight to the operating field. Therefore, the patient's neck needs to be brought into a hyperextended position. In a considerable number of patients, this is not possible due to anomalies in the cervical spine anatomy or soft tissue scarring, e.g., after radiation. In these cases, adequate visualization of relevant laryngeal structures cannot be ensured using a conventional rigid operating laryngoscope, which may negatively affect the outcome of these patients.

**Methods:**

We present a system based on a 3D-printed prototype of a curved laryngoscope with three integrated working channels (sMAC). The curved profile of the sMAC-laryngoscope is specifically adapted to the nonlinear anatomy of the upper airway structures. The central working channel provides access for flexible video endoscope imaging of the operating field while the two remaining channels provide access for flexible instrumentation. In a user study (*n* = 11), visualization and reachability of relevant laryngeal landmarks as well as the feasibility of basic surgical procedures with the proposed system were examined in a patient simulator. In a second setup, the system was evaluated for its applicability in a human body donor.

**Results:**

All participants of the user study were able to visualize, reach and manipulate the relevant laryngeal landmarks. Reaching those took significantly less time in the second attempt compared to the first one (27.5 s ± 5.2 s vs. 39.7 s ± 16.5 s, *p* = 0.008) indicating a significant learning curve for handling the system. Instrument changes were performed quickly and reliably by all participants (10.9 s ± 1.7 s). All participants were able to bring the bimanual instruments into position for a vocal fold incision. Relevant laryngeal landmarks could be visualized and reached in the human body donor setup.

**Conclusion:**

Possibly, the proposed system may develop into an alternative treatment option for patients with early-stage laryngeal cancer and restricted mobility of the cervical spine in the future. Further improvements of the system could include finer end effectors and a flexible instrument with a laser cutting tool.

## 1. Introduction

Laryngeal cancer is one of the most common tumors in the head and neck area. In 2020 an estimated 184,615 new cases of laryngeal cancer were diagnosed worldwide and 99,840 patients died from the disease (1). Treatment options for patients with laryngeal cancer include radiation-based as well as surgical approaches. In the last decades, transoral laser microsurgery (TLM) and exclusive radiotherapy emerged as the preferred treatment modalities for patients with early-stage laryngeal cancer (T1–T2) (2–4). Compared to open partial laryngectomy, TLM offers shorter hospitalization times and faster return of postoperative swallowing, while showing comparable oncologic results (5, 6). Advantages of TLM over radiotherapy in the treatment of early glottic cancer include a shorter treatment time and improved laryngeal preservation (7–11).

One of the main limitations of TLM is the laser-beam requiring a straight line of sight through the oral-oropharyngeal corridor into the operating field. Therefore, adequate exposure and visualization of laryngeal structures is crucial for an effective intervention (12). To achieve satisfactory exposure of the glottic plane, the cervical spine of the patient needs to be brought into a hyperextended position. In an estimated 10%–20% of the patients, adequate exposure of the glottic plane is not possible because of restricted mobility of the cervical spine, trismus or substantial scarring of neck tissue after radiation (13, 14). Especially the indication of TLM for tumors involving the anterior commissure remains critical in some cases due to difficulties in achieving good exposure of this region, which may lead to incomplete resection of the tumor and inferior oncologic outcomes (15–17). Furthermore, in TLM, considerable forces are applied to the maxillary incisors and laryngopharynx by the straight and rigid microlaryngoscope (18). These forces may cause postoperative complications including transient laryngeal edema, hematoma, hypoglossal palsy, taste alteration, dysphagia or dental injuries in a substantial portion of patients that undergo microlaryngoscopy (19, 20).

To also offer ideal surgical treatment to patients with restricted mobility of the cervical spine, surgical systems have to be adapted to the nonlinear anatomy of the laryngopharyngeal region. The former Flex robotic system (Medrobotics, Raynham, United States) met these requirements by establishing a computer-operated flexible endoscope to access the surgical site. The Da Vinci Single Port system (Intuitive Surgical, Sunnyvale, United States) offers similar capabilities, but it takes a different approach by using instruments and optics that are semi-flexible through joggle-joints. Cadaver studies and first clinical reports show promising results for the treatment of supraglottic pathologies with these systems (21–24). However, particularly high costs and complex handling of these systems compared to TLM inhibit their widespread adoption in clinical practice (25, 26).

Our research group has proposed a system (sMAC) for laryngeal surgery based on a curved video-laryngoscope, which is equipped with flexible instruments to access the operating site in a nonlinear manner. We demonstrated the capability of the system to visualize and manipulate laryngeal structures in a porcine larynx model as well as in a human cadaver study (27, 28). Additionally, we demonstrated in a preclinical study that our system applies significantly less force on the upper front teeth and laryngopharynx compared to direct rigid microlaryngoscopy used in TLM (29). However, the video laryngoscope is designed for intubation procedures and not surgical purposes. Therefore, one of the main limitations of the system is the camera unit. The image quality is subpar compared to other commercially available endoscopes and zoom capabilities are lacking. Moreover, the whole system is composed of several subcomponents that need to be assembled before use.

To overcome these limitations, we developed a next-generation sMAC system, which is based on a 3D-printed curved laryngoscope with integrated working channels. One of the working channels provides access for a flexible video-endoscope for visualization of the operating field. The two remaining channels provide access for flexible instrumentation. The goal of this user study is to assess the capabilities of this improved system regarding visualization as well as the manipulation of laryngeal structures in a patient simulator as well as a human body donor.

## 2. Materials and methods

### 2.1. 3D-printed curved laryngoscope

#### 2.1.1. Design

**Figure 1:**
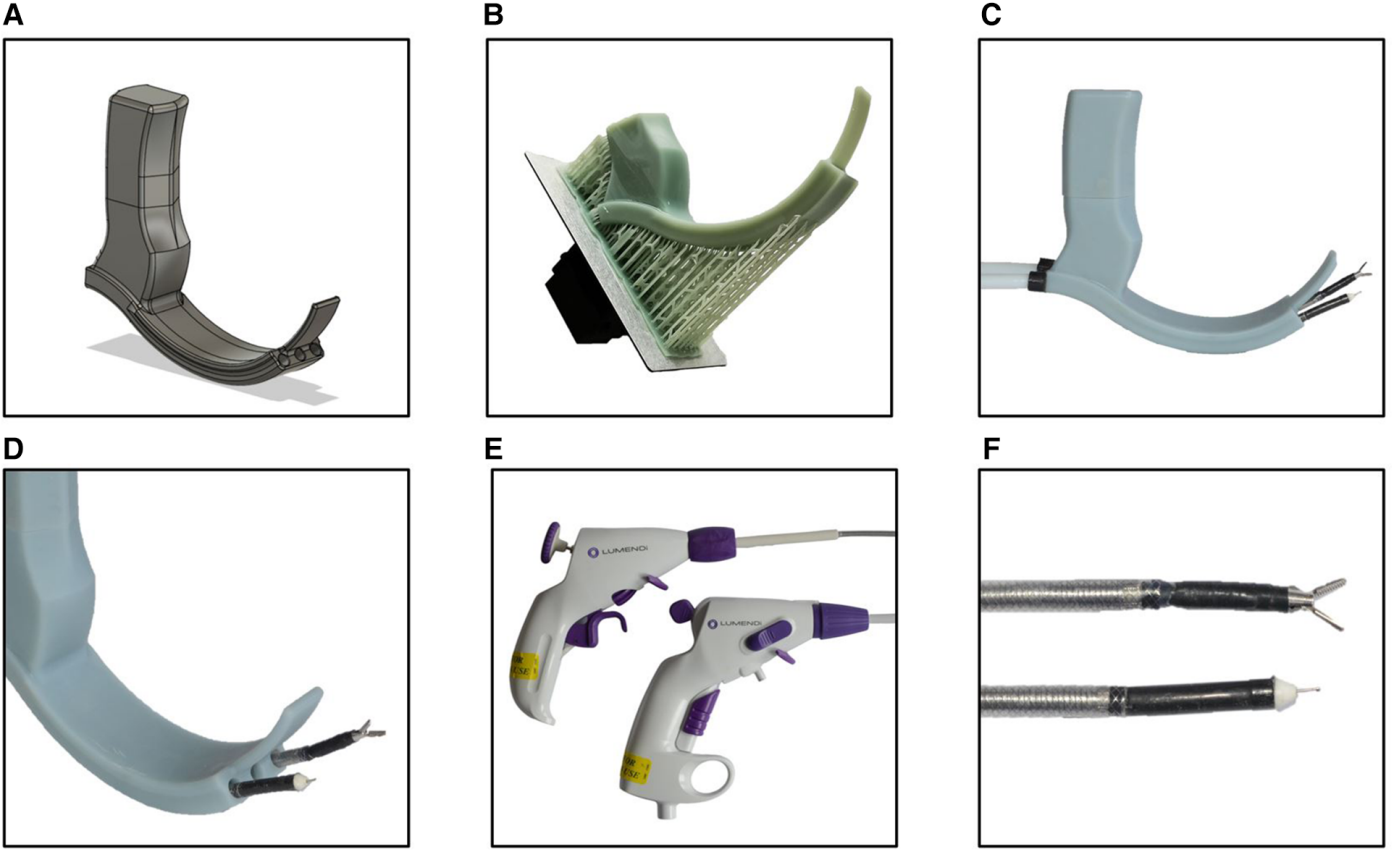
(**A**) CAD model of the sMAC laryngoscope with integrated working channels. (**B**) 3D-printed blade of the laryngoscope with support structures still attached. (**C,D**) Assembled 3D-printed sMAC laryngoscope with flexible instruments introduced to the working channels. (**E,F**) Control units and instrument tips of the fully flexible surgical instruments.

The sMAC-laryngoscope with its hyperangulated shape, shown in Figures 1A–D, is specifically designed to follow the nonlinear anatomy of the upper aerodigestive tract. It features three working channels with a diameter of 6.5 mm to provide access to the operating site for instrumentation and a flexible video endoscope. The grip of the laryngoscope with its hexagonal shape is designed to match the profile of the mounting clamp.

#### 2.1.2. 3D-printing process

Because of the complex shape and cavities of the design, conventional fused deposition modeling (FDM) printing processes were not suitable to manufacture a functional prototype. Instead, the prototype was manufactured in an LCD printing process with an LD-002H (Creality, Shenzen, China) resin 3D-printer. Because the size of the prototype surpassed the size of the printing bed, the blade and the grip of the laryngoscope were printed separately and assembled later. One of the drawbacks of most commercially available desktop resins are their brittleness and general poor mechanical properties. To enable the prototype to withstand the forces applied during surgery, it was printed with a special high strength engineering resin (Build Resin, Siraya Tech, San Gabriel, United States). After printing (Figure 1B) support structures were removed, the parts were then washed with alcohol and cured under ultraviolet light.

### 2.2. Flexible video endoscope

To depict the operating field, the flexible video endoscope 11101HDK (Karl Storz, Tuttlingen, Germany) was used. It has a 30 cm working length and an outside diameter of 3.7 mm. The camera located in the tip features high-definition image quality and a 100° angle of view. Moreover, the tip of the endoscope allows up/down deflection of 140°. The image of the flexible endoscope was displayed on an 18.5-inch TP101 monitor (Karl Storz) with a resolution of 1920 × 1080 pixels.

### 2.3. Instrumentation

To access the operating field through the curved path of the working channels, fully flexible instruments are required. We used flexible instruments of the DiLumen C^2^ system (Lumendi, Westport, United States), depicted in Figures 1E,F, which were originally developed for endoluminal surgery of the digestive tract. These manually operated, single-use instruments have an outside diameter of 6 mm. The manufacturer provided us with instruments with a reduced working length of 55 cm compared to the standard working length used in endoluminal colorectal surgery. The DiLumen I_g_ grasper (Lumendi) features 6 mm long jaws with an opening angle of 60°. The tip of the grasper is 90° deflectable in two degrees of freedom resulting in a working space that corresponds to one half of a spherical shell. Besides the grasper instrument we also used the DiLumen I_k_ (Lumendi) monopolar electrosurgical knife in our experiments. The instrument comes with a 4 mm long extendable blade and allows deflection of the instrument tip up to 90° in one degree of freedom.

### 2.4. Experimental setup

**Figure 2:**
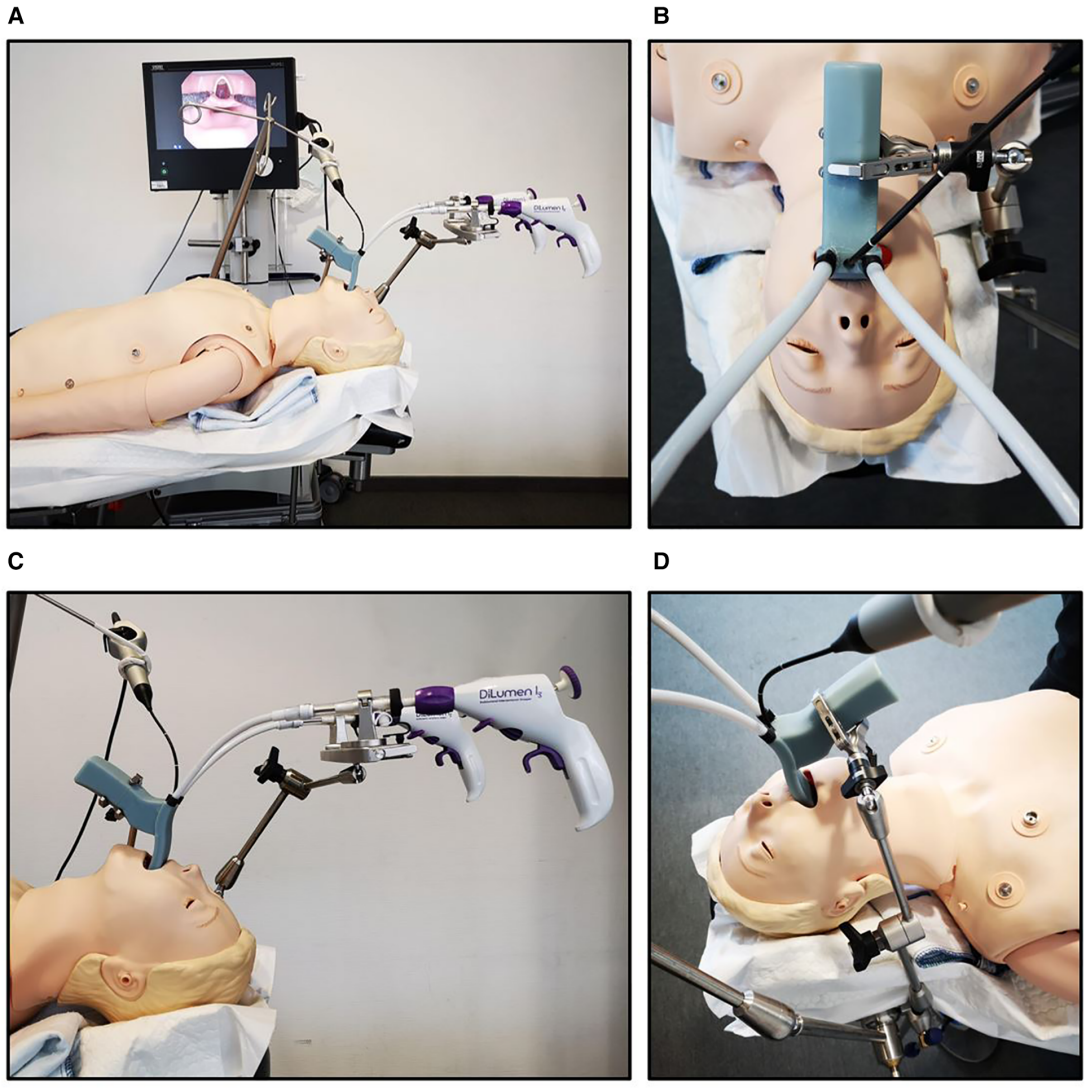
(**A–D**) Experimental setup of the user study. After the patient simulator was placed on the operating table, the 3D-printed sMAC laryngoscope was attached to the operating table using a clamping jaw and an articulated stand. The image of the flexible video endoscope was displayed on an 18.5-inch Monitor.

For our experiments, first a true-to-life Resusci Anne patient simulator (Laerdal, Stavanger, Norway) and then a human fresh-frozen body donor were placed on an operating table. The 3D-printed sMAC laryngoscope was attached to the operating table using an articulated stand (28272 HA, Karl Storz) in combination with a clamping jaw (28272 UFN, Karl Storz). The metal instrument holder (Lumendi) located at the head of the operating table supported the distal proximal ends of the instruments and allowed the surgeon simultaneous use of both instruments. The flexible endoscope was placed in the central working channel for visualization. The prototype was inserted into the oral cavity of the patient simulator. After ensuring correct positioning and full laryngeal exposure, the articulated stand was fastened, and the system locked into place (Figure 2).

### 2.5. User study on a patient simulator

**Figure 3:**
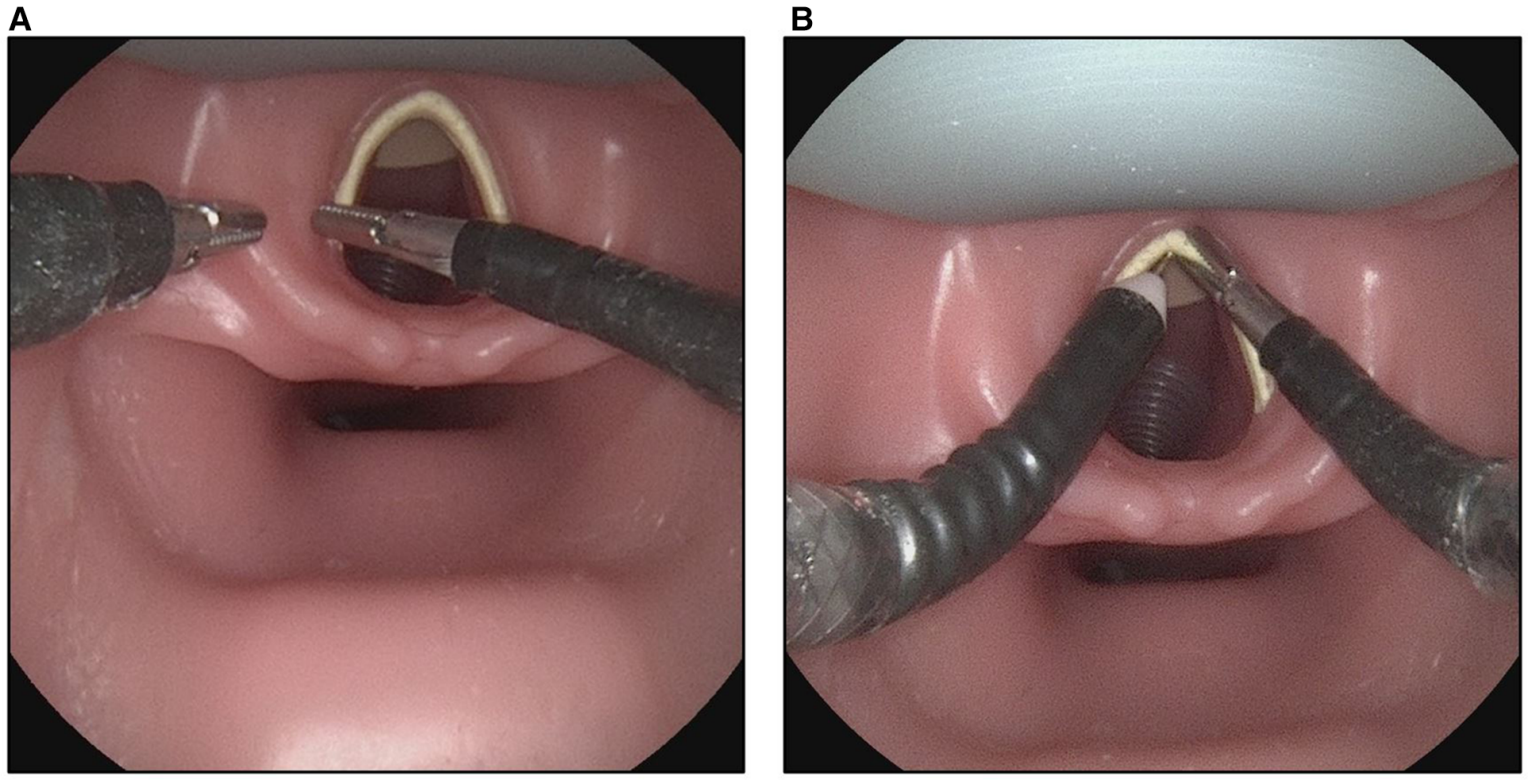
Photographic documentation of the user study. (**A**) Participant of the study reaching for the left vestibular fold as part of the first task. (**B**) Flexible instruments brought into position to cut the vocal fold.

The user study is comprised of 11 participants, namely four medical students in the clinical part of their training and seven residents of the ENT-department. All participants evaluated the sMAC system regarding visualization and manipulation of laryngeal structures on the patient simulator according to the following study protocol: The participants were asked to reach for a series of anatomical landmarks in the larynx with one of the grasper instruments (Figure 3A). The anatomical landmarks were the left and right vocal fold, the left and right vestibular fold, the anterior commissure, the postcricoid region and the ventral subglottic region. After completing this task, each participant carried out the procedure a second time, again reaching for the described landmarks. Afterwards the participants were asked to remove one of the grasper instruments from its working channel and exchange it for the electrosurgical knife instrument. In the final task the participants were directed to reach for the vocal fold with the grasper instrument in one hand and simulate a cut next to the grasper with the electrosurgical knife in the other hand (Figure 3B). The time needed to complete each task was measured and photo documentation was carried out.

### 2.6. Application in a human body donor

**Figure 4:**
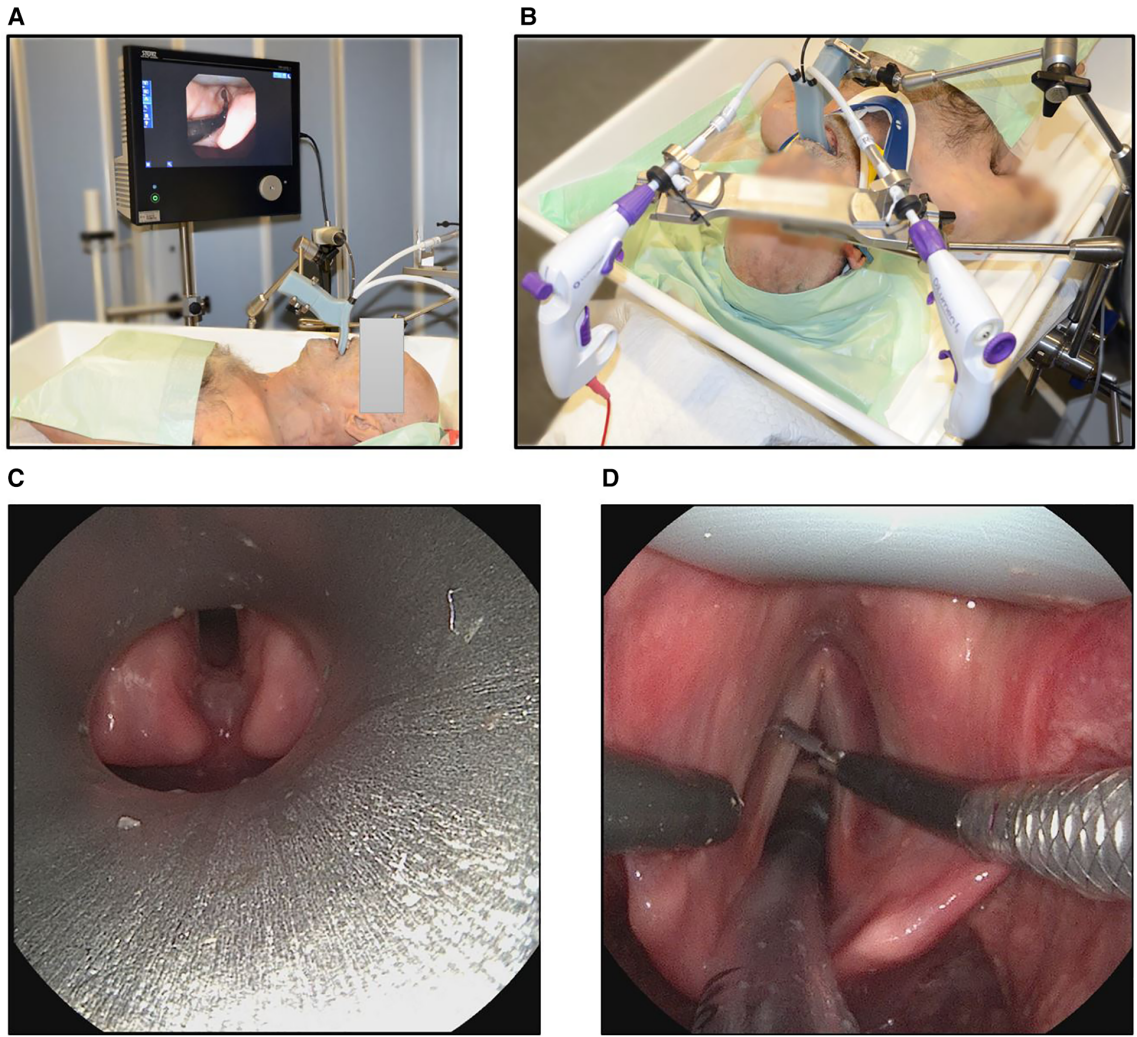
Experimental setup of the sMAC on a human body donor torso. The sMAC laryngoscope was attached to the operating table using a clamping jaw and an articulated stand. The image of the flexible video endoscope was displayed on an 18.5-inch Monitor as in the user study. (**A**) Mobile and (**B**) immobilized neck *via* cervical support. (**C**) View of the glottic plane with an conventional operating laryngoscope positioned by an experienced head and neck surgeon and with the neck of the body donor not being immobilized. (**D**) View of the operating field with the sMAC system in a human body donor with immobilized neck. The grasper instrument is on the right side, the monopolar needle instrument on the left side and an endotracheal tube is inserted to the trachea.

In a second setup with the same configuration, the system was used on a human fresh-frozen body donor. In this case, two scenarios were distinguished, once with and without cervical support (*Stifneck*, Laerdal, Stavanger, Norway). An experienced head and neck surgeon was then asked to position a widely used Kleinsasser operating laryngoscope (OP292, Aesculap, Tuttlingen, Germany) and show the glottic plane. Afterwards the surgeon was asked to adjust the sMAC system to the vocal fold level and reach for relevant laryngeal landmarks (left and right vocal folds, plicae vocales, anterior commissure, postcricoid region, subglottic space) with the grasping instrument (Figures 4A,B). The experiments involving human body donors were approved by the local ethics committee (# 89/19).

### 2.7. Statistics

Statistical analysis was conducted with the help of MATLAB and the included statistics and machine learning toolbox (MathWorks, Natick, United States). The measured times were checked for normality using the Shapiro-Wilk test. To compare the results of the first trial of reaching for laryngeal landmarks to the second trial, a paired sample *t*-test was performed. The differences between the group of medical students compared to the group of residents were evaluated using a two-sample *t*-test. Statistical significance was considered for *p*-values <0.05. Results are given in the form mean ± standard deviation unless stated otherwise.

## 3. Results

Positioning of the sMAC prototype was fast and simple using the mounting system described above. The mounting system held the prototype in place during the entire course of the experiments with no readjustment necessary. The flexible video endoscope delivered a clear image, which was satisfactory for surgical purposes. Visualization of all the relevant laryngeal landmarks was always possible. Furthermore, specific regions of the glottic plane could be inspected closer by bringing the endoscope camera closer to the region of interest. All participants of the user study were able to handle the instruments with the system controls.

**Figure 5:**
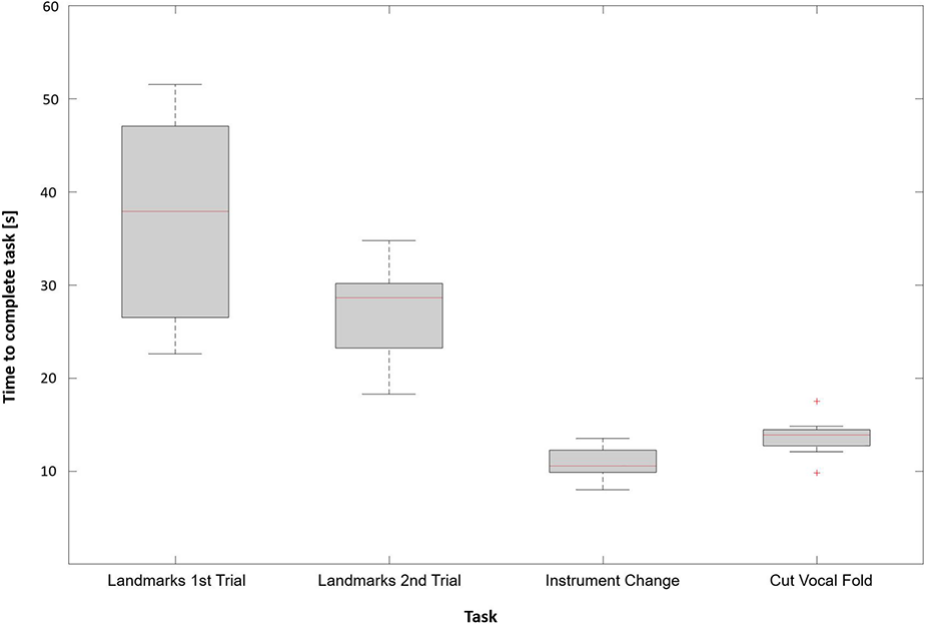
Time required by the participants to complete each task.

**Figure 6:**
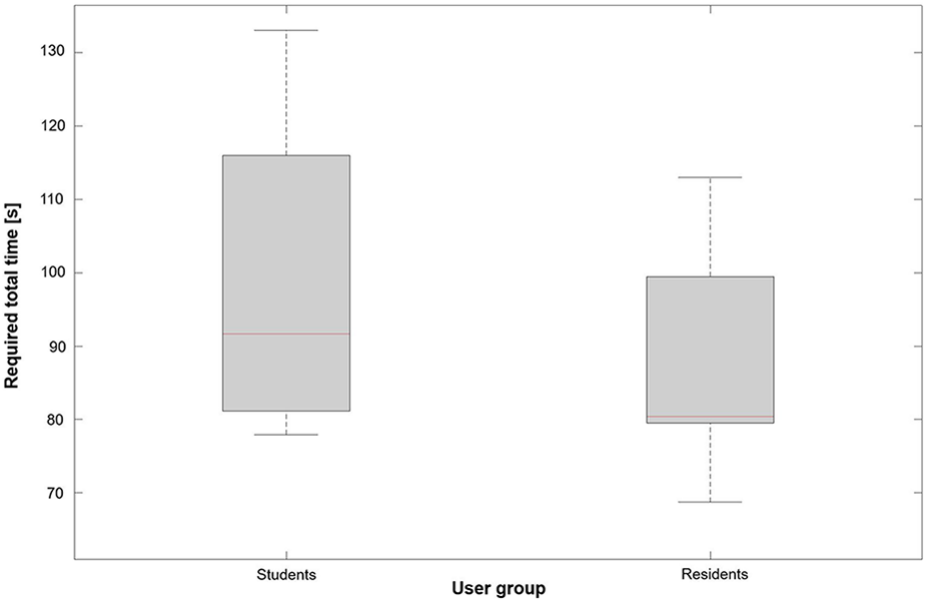
Required total time needed to complete the tasks for each user group.

All participants were able to complete the given tasks successfully. The time the participants needed to perform each given task is illustrated in Figure 5. Reaching for the laryngeal landmarks took significantly less time in the second trial as compared to the first trial (27.5 s ± 5.2 s vs. 39.7 s ± 16.5 s, *p* = 0.008). Instrument changes were performed in a time of 10.9 s ± 1.7 s. To bimanually perform a cut in the vocal fold the participants needed 13.7 s ± 1.9 s. On average the group of residents took less time to complete the given tasks compared to the group of medical students (88.0 s ± 15.3 s vs. 98.6 s ± 24.6 s, *p* = 0.48), as shown in Figure 6. However, this result was not statistically significant.

We could also compare the accessibility of laryngeal structures with the sMAC system to a conventional operating laryngsocope. The experienced head and neck surgeon was not able to show the glottic plane with a conventional operating laryngoscope, when the spine of the body donor was immobilized. With a mobile cervical spine the surgeon was able to show the posterior parts of the glottic plane. However he was not able to show the anterior parts including the anterior commissure (see Figure 4C), even when considerable forces were applied. In contrast the surgeon was able to insert the sMAC prototype without any difficulty with both mobile and immobile cervical spine of the body donor and to specifically target the landmarks relevant for laryngeal surgery using the gripping instrument. An endotracheal tube inserted into the trachea of the body donor did not significantly obstruct the view and accessibility of the operating field, as shown in Figure 4D.

## 4. Discussion

The current state of the art surgical treatment method of early-stage laryngeal cancer is considered to be TLM (30). Moreover, transoral robotic surgery (TORS) with the Da Vinci robotic system (Intuitive Surgical) has emerged as an established treatment method especially in oropharyngeal and supraglottic lesions (31). The (off-label) treatment of the glottis is still rather in its early stages. Nevertheless, several case-studies suggest safe use with comparable results and outcomes in this instance as well (32, 33). However, both methods, TLM and TORS, are limited by the prerequisite to access the operating site in a straight path. This is especially difficult regarding lesions located at the anterior commissure and in patients with restricted mobility of the neck (34–36). Regarding TORS, there are currently no large randomized multicentric trials which unanimously support the advantage of TORS over TLM in the treatment of laryngeal cancer to justify the higher costs associated with TORS (25, 37). In approximately 10%–20% of the patients, it is not possible to achieve adequate exposure of the entire larynx by direct microlaryngoscopy. Therefore, a considerable number of patients are not suitable for TLM (38). Furthermore, in TLM, forces which are applied to the maxillary incisors and laryngopharynx by the rigid microlaryngoscope may cause postoperative complications including transient laryngeal edema, hematoma, hypoglossal palsy, taste alteration, dysphagia or dental injuries (19, 39).

The sMAC system developed by our research group uses a curved video laryngoscope with added flexible instruments to follow the nonlinear anatomy of the upper airways. With this system we could show the feasibility of visualization and manipulation of laryngeal structures in a porcine model as well as a human body donor (27, 28). Additionally, we showed in a preclinical study that the curved sMAC system applies significantly less force on the upper front teeth and laryngopharynx compared to direct microlaryngoscopy used in TLM which may reduce the occurrence of previously described complications (29). Compared to the electro-mechanical separation of the Da Vinci system, the continuous flexible instruments provide the surgeon with tactile feedback of the manipulated tissue (28). However, one of the main limitations of the sMAC system is the image quality of the video laryngoscope, which was designed for intubation procedures and not for surgical interventions. Moreover, the camera of the video laryngoscope is in a fixed position, requiring the entire system to be repositioned to change the centre of the image. Because the end effector of the flexible grasper instrument was originally designed for surgery of the digestive tract, it is too large for the manipulation of fine laryngeal structures (27). Furthermore, the design of the flexible instruments could limit targeted work at specific angles. However, this also applies to TORS and to classic TLM due to the narrow spatial conditions in the oral-oropharyngeal corridor. Another point is that the flexible instruments used might not be able to exert the same high contact pressure on the grippers due to their design compared to classic TLM or TORS. However, this effect did not restrict the handling of the system in our studies.

The 3D-printed second-generation sMAC laryngoscope with integrated working channels presented in this study has a curved shape adapted to the nonlinear path of the oral-oropharyngeal corridor. It allows the use of arbitrary flexible video endoscopes with diameters up to 6 mm. The endoscope used in this study offers high-definition image quality suitable for surgical purposes. Moreover, the deflectable tip of the endoscope enables the surgeon to inspect areas of interests up close as well as from different angles. The correct positioning of the system with the clamping jaw and the articulated stand is straightforward and firmly locks the system into place. The visualization and surgical manipulation of laryngeal structures with the prototype system was demonstrated in a true-to-life patient simulator. The participants took significantly less time to reach for the laryngeal landmarks in their second trial compared to their first trial, which indicates a steep learning curve for handling the system. The performance of the group of medical students compared to the group of residents showed no statistically significant difference. Whereas the sMAC system of the first generation consists of several subcomponents that need to be assembled before use, the 3D-printed sMAC laryngoscope is one integrated part. During the entire course of the experiments, the 3D-printed prototype showed no signs of any mechanical failure. Due to the rapidly developing 3D printing industry the cost of 3D printers as well as printing materials for consumers decreased considerably. Consumer grade LCD 3D printers like the one used in this study to print the prototype laryngoscope are available for less than 300 USD. The material cost to print one laryngoscope with the engineering resin used to print the prototype is approximately 10 USD. Therefore, the main cost factor of a procedure with the presented system is the acquisition cost of the flexible video endoscope and the cost of the single use flexible instruments. The negligible material and printing costs enable the possibility of manufacturing custom patient specific solutions adapted to individual anatomic conditions in the future.

A system comparable to the demonstrated sMAC system is the Flex system by Medrobotics. In our opinion, both systems have their advantages, which are listed below. Advantages of the Flex system: (I) ability to change the position of the endoscope during surgery; (II) proven clinical suitability in clinical studies; (III) system and instruments are produced by the same manufacturer. Advantages of the sMAC system: (I) higher stability due to its rigid construction; (II) lower financial burden due to its lower content of technology; (III) the flexible endoscope can be advanced over the tip of the demonstrator to visualize remote areas of the surgical field; (IV) potentially re-usable components.

Nevertheless, the presented sMAC system of the second generation has several limitations. Firstly, the engineering resin used to print the prototype laryngoscope presented in this study, is not FDA approved for the human use. There are alternative resins (BioMed Clear Resin, Formlabs, Somerville, United States) that are FDA approved for applications with skin or mucosal membrane contact. However, these resins are more expensive which would increase the material costs for the 3D-printed laryngoscope to approximately 50 USD. Secondly, as the jaws of the grasper instrument were initially designed for colorectal surgery, they are too large, and they should be adapted to the size of the vocal folds. At present, handling of the sMAC for one person is somewhat complex and therefore requires the surgeon to interrupt surgery to reposition the flexible endoscope. In the future, in a next-generation demonstrator, the fixation of the flexible endoscope will be integrated into the sMAC. Finally, there is no option yet for using a laser as a cutting tool in the 3D-printed sMAC system. As the CO_2_-laser is the preferred cutting tool in TLM, this should also be considered in non-linear larynx surgery. While there is a flexible monopolar electrosurgical knife instrument available, these monopolar cutting tools can cause thermal injuries and carbonization of the laryngeal mucosa (35, 40). To be able to safely apply such techniques *in vivo*, the prototype may be equipped with an additional working channel for plume evacuation in the future. Also fogging of the tip of the video-endoscope due to condensing water vapor on the cold endoscopic lens could be a problem in actual patient care. We will therefore consider physical and chemical anti-fog methods and apply them to the prototype soon.

## 5. Conclusion

In this study we demonstrated the capabilities of the proposed system consisting of a curved 3D-printed sMAC laryngoscope, a flexible video endoscope and flexible instrumentation to visualize and manipulate relevant laryngeal structures in a patient simulator and a human body donor. While preserving the described advantages of the first generation sMAC system the presented second-generation system additionally offers more flexible visualization, a reduced complexity, and the possibility to create custom solutions adapted to the anatomy of the patients' upper airways. To further evaluate the feasibility of laryngeal surgery with the presented prototype system, a detailed human cadaver study is being prepared. Further improvements of the system could include adaption of the flexible grasper instruments and inclusion of a laser cutting option.

## Data Availability

The raw data supporting the conclusions of this article will be made available by the authors, without undue reservation.
